# A new prototype melt-electrospinning device for the production of biobased thermoplastic sub-microfibers and nanofibers

**DOI:** 10.1186/s40824-019-0159-9

**Published:** 2019-03-29

**Authors:** Kylie Koenig, Konrad Beukenberg, Fabian Langensiepen, Gunnar Seide

**Affiliations:** Aachen-Maastricht Institute for Biobased Materials (AMIBM), Maastricht University, Brightlands Chemelot Campus, Urmonderbaan 22, 6167 RD Geleen, The Netherlands

**Keywords:** Fiber spinning, Nanotechnology, Polylactic acid, Nanofiber nonwoven, Eco-friendly production, Melt spinning, Fiber production, Electrospinning, Process development

## Abstract

Sub-microfibers and nanofibers have a high surface-to-volume ratio, which makes them suitable for diverse applications including environmental remediation and filtration, energy production and storage, electronic and optical sensors, tissue engineering, and drug delivery. However, the use of such materials is limited by the low throughput of established manufacturing technologies. This short report provides an overview of current production methods for sub-microfibers and nanofibers and then introduces a new melt-electrospinning prototype based on a spinneret with 600 nozzles, thereby providing an important step towards larger-scale production. The prototype features an innovative collector that achieves the optimal spreading of the fiber due to its uneven surface, as well as a polymer inlet that ensures even polymer distribution to all nozzles. We prepared a first generation of biobased fibers with diameters ranging from 1.000 to 7.000 μm using polylactic acid and 6% (*w*/w) sodium stearate, but finer fibers could be produced in the future by optimizing the prototype and the composition of the raw materials. Melt electrospinning using the new prototype is a promising method for the production of high-quality sub-microfibers and nanofibers.

## Introduction

Nanotechnology can be generally defined as the development, handling and control of structures or materials with at least one dimension within the size range 1–100 nm, and the advent of precision tools for nanoscale engineering has promoted great interest in this emerging field over the last 30 years [1, 2]. Nanotechnology exploits the properties of materials that depend on size or structure, particularly properties that differ from the behavior of individual atoms/molecules or larger masses of the same material [2]. The term “nanofiber” is frequently used in the literature to describe very thin fibers without a specified size limit, but a stricter definition as used by the Deutsches Institut für Normung (DIN) standard among others is a structure with two external nanoscale dimensions and a third external dimension that is considerably larger than the nanoscale [3]. However, a comparison of many studies shows that the same term is often used as soon as the fiber diameter falls below 1 μm [4]. Although such fibers are not nanofibers according to the DIN standard, the designation has become established and consolidated in recent years. With respect to the DIN standard, another term used to describe fibers with a diameter in the hundreds of nanometers range is “sub-microfiber”.

**Fig. 1:**
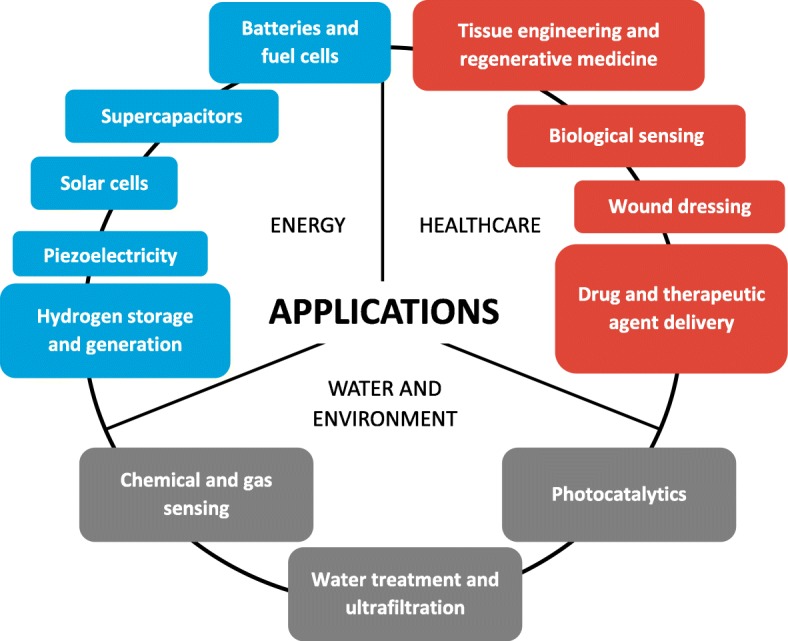
Applications of sub-microfibers and nanofibers

The small diameter of sub-microfibers and nanofibers provides a high surface-to-volume ratio while maintaining or even improving flexibility compared to conventional fibers. Additionally, many production methods yield porous fibers thus increasing the surface area even further [5]. These properties make such fibers extremely versatile. Their diverse applications include air and water filtration [6, 7], the separation of water/oil and air/oil mixtures [4, 8], technical uses such as the development of lithium-air batteries [9–12], optical sensors [13] and textiles [5, 14], and medical applications such as tissue engineering [15–23], drug delivery [18, 19, 24–36], and the diagnosis and treatment of cancer [27, 36–40] (Fig. 1) [41]. The applications of sub-microfibers and nanofibers depend on their physical and mechanical properties, which in turn depend on the manufacturing process. This short report provides an overview of current production methods before describing a novel and scalable melt-electrospinning prototype device and its deployment for the processing of biobased materials into fibers.

## Current manufacturing processes for sub-microfibers and nanofibers

**Fig. 2:**
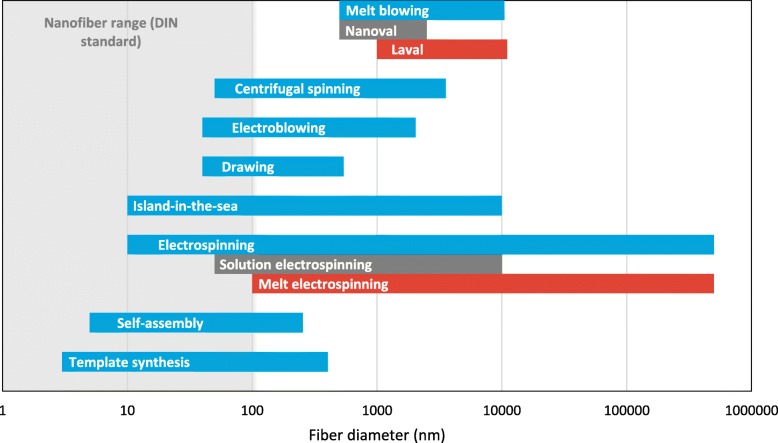
Nanofiber production technologies and the corresponding fiber diameter range: melt blowing [4], nanoval [42, 43], Laval [42], centrifugal spinning [44], electroblowing [45], drawing [46], island-in-the-sea [47], electrospinning [4], solution electrospinning [4], melt electrospinning [4], self-assembly [48, 49], template synthesis [50]

Sub-microfibers and nanofibers can be produced from a range of biomaterials, such as polysaccharides (e.g. chitosan, cellulose, or alginate) and proteins (e.g. gelatin, keratin, or collagen), as well as synthetic polymers, such as polycaprolactone (PCL), polyurethane (PU), polylactic acid (PLA), and poly(lactic-*co*-glycolic) acid (PLGA). Figure 2 provides an overview of current major nanofiber production technologies and the fiber diameters that have typically been achieved using those methods. Only the most common processes are mentioned and there are many variants of these methods that we do not discuss in detail [42–50].

**Fig. 3:**
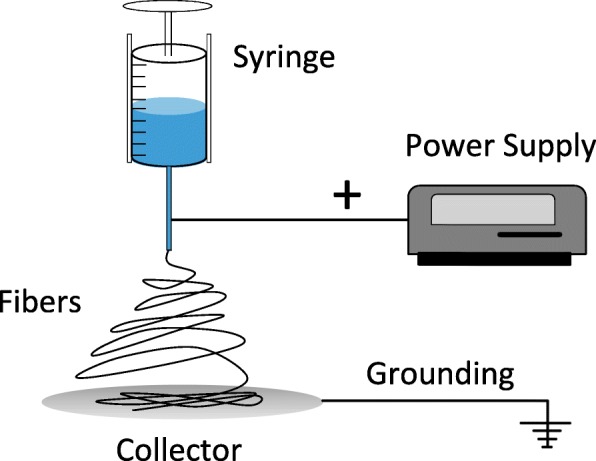
Basic setup of the electrospinning production method

Electrospinning is the most common production method for sub-microfibers and nanofibers, and two fundamental techniques can be distinguished: solution electrospinning and melt electrospinning. Electrospinning combines a strong electrostatic field with the principle of Taylor cone formation. When a droplet of a liquid becomes charged in a field of sufficient strength, the electrostatic repulsion is strong enough to overcome the surface tension and the droplet is stretched. If the charge reaches a certain threshold, a jet erupts from the liquid droplet and this is known as a Taylor cone [51–53]. If the liquid is viscous and cohesive, the jet does not break up into droplets (the principle of electro-spraying) but forms an electrically charged laminar jet, which elongates due to electrostatic repulsion. The jet dries (in the case of solution electrospinning) or cools sufficiently to become solid (in the case of melt electrospinning) and a nanoscale fiber is produced [54]. The basic setup for electrospinning is shown in Fig. 3. Solution electrospinning is used more frequently than melt electrospinning for the production of nanofibers because a smaller fiber diameter can be achieved (high hundreds of nanometers), and the equipment has a simpler design and higher productivity compared to current melt electrospinning devices [55]. The finest fiber produced by melt electrospinning thus far was 80 nm in diameter [56], although this in not yet routine and typically the fiber diameter is > 2 μm [45]. The major advantage of melt electrospinning is that it does not require a solvent, avoiding any risk of toxic solvents being carried over into the mature fiber [57]. Electrospinning is compatible with many different polymers and multiple applications (Table 1).

**Table 1:** Overview of electrospinning methods, typical fiber diameters and applications

Publication	Production method	Polymer	Fiber diameter [nm]	Application
Wang 2002 [12]	Solution	Poly(acrylic acid) − poly(pyrene methanol)	100–300	Optical sensors
Li 2012 [87]	Melt (laser melt)	Poly(L-lactic acid)	2000–7000	Biomedical
Dalton 2006 [89]	Melt	Poly(ethylene glycol) and poly(epsilon-caprolactone)		
Yoon 2013 [90]	Melt and S/M-hybrid	Silk fibroin and poly(L-lactic acid)	PLA: 8900 SF: 820	Biomedical (scaffolding)
Zhou 2006 [91]	Melt	Poly(L-lactic acid)		Filtration
Kim 2010	S/M hybrid	Poly(lactic-*co*-glycolic acid)	2800 (S) 530 (M)	Biomedical (scaffolding)
Scholten 2011 [7]	Solution	Polyurethane	Low 1000s	Air filtration (removal of volatile organic compounds

**Fig. 4:**
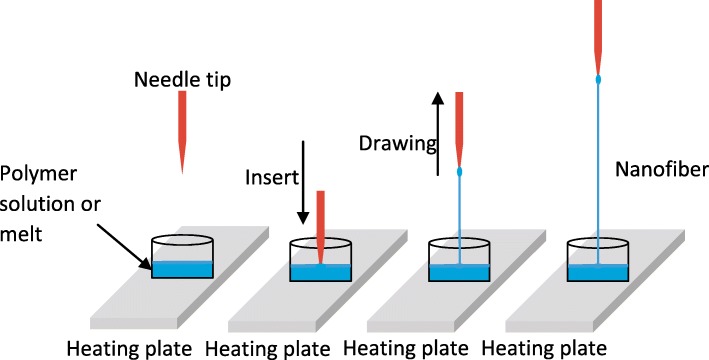
Basic setup of the fiber-drawing production method

Another common method for nanofiber production is fiber drawing. Here, a solid tip is placed in contact with a liquid polymer and then drawn away, leaving behind a string of polymer liquid that solidifies into a fiber (Fig. 4). Like electrospinning, this method is compatible with polymer melts [58] and polymer solutions [46]. One of the main advantages of this method is that it allows the evaluation of single fibers [59]. Drawing typically produces narrower fibers than electrospinning, with diameters of tens of nanometers (Table 2).

**Table 2:** Overview of non-electrospinning methods, typical fiber diameters and applications

Publication	Production method	Polymer	Fiber diameter [nm]	Application
Xing 2008 [58]	Drawing	Poly(trimethylene terephthalate)	60	Optical fibers
Ma 2016 [46]	Drawing	Polyethylene	40	
Nakata 2007 [61]	Island in the sea	Polyamide 6/poly(ethylene terephthalate)	39	
Uppal 2012 [63]	Melt-blown		290	Filtration
Luo [97]	Template synthesis	Silver/cross-linked poly(vinyl alcohol)	Sub-micro	Various
Wang 2010 [98]	Template synthesis	Carbon	20	Various
Rolandi 2014 [99]	Self-assembly	Chitin	various	Various
Xu 2017 [100]	Self-assembly	Polypeptide		Drug delivery
Hammami 2014 [74]	Centrifugal	Polyamide 6	200–800	Various

**Fig. 5:**
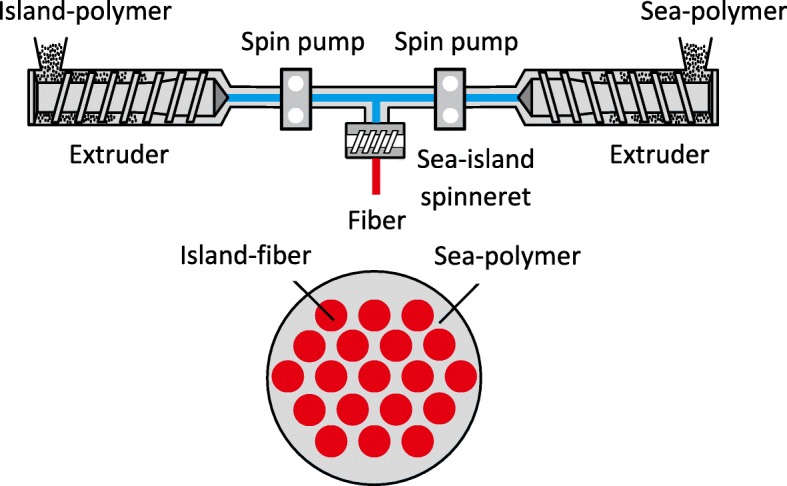
Island-in-the-sea nanofiber production method

The island-in-the-sea method (Fig. 5) is a subtype of conventional melt spinning, but two different polymers are involved. One of them (the sea polymer) is spun into a thick fiber within which multiple thinner fibers of the other (the island polymer) are suspended. Following primary extrusion, the sea polymer is removed to leave the nanoscale island-polymer fibers behind [60]. This method has been used to create polyamide 6/polyethylene terephthalate nanofibers with a consistent diameter of 39 nm [61].

**Fig. 6:**
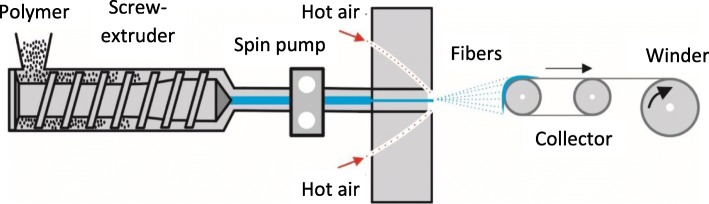
Basic setup of the melt-blowing production method

Melt-blown fibers are produced by extruding a polymer melt through small nozzles surrounded by high-speed flowing gas, typically resulting in microfibers approximately 2 μm in diameter (Fig. 6). However, individual sub-microfibers/nanofibers with diameters of ~ 100 nm have been produced using an ideal setup comprising an annual air die, Finaplas polypropylene (PP) with a melt flow rate (MFR) of 35 as the polymer, a polymer temperature of 290 °C, a gas temperature of 400 °C and a feed rate of 4.11·10^− 6^ kg/s [62]. Like electrospinning, which can be scaled up by multiplying the number of jets, melt-blowing can be scaled up by multiplying the number of nozzles to reduce costs [63]. However, unlike electrospinning, which can produce aligned fibers, melt-blown fibers are deposited randomly into non-woven sheets. These are particularly suitable for filtration applications, but melt blowing cannot be used for applications that require oriented fiber sheets.

**Fig. 7:**
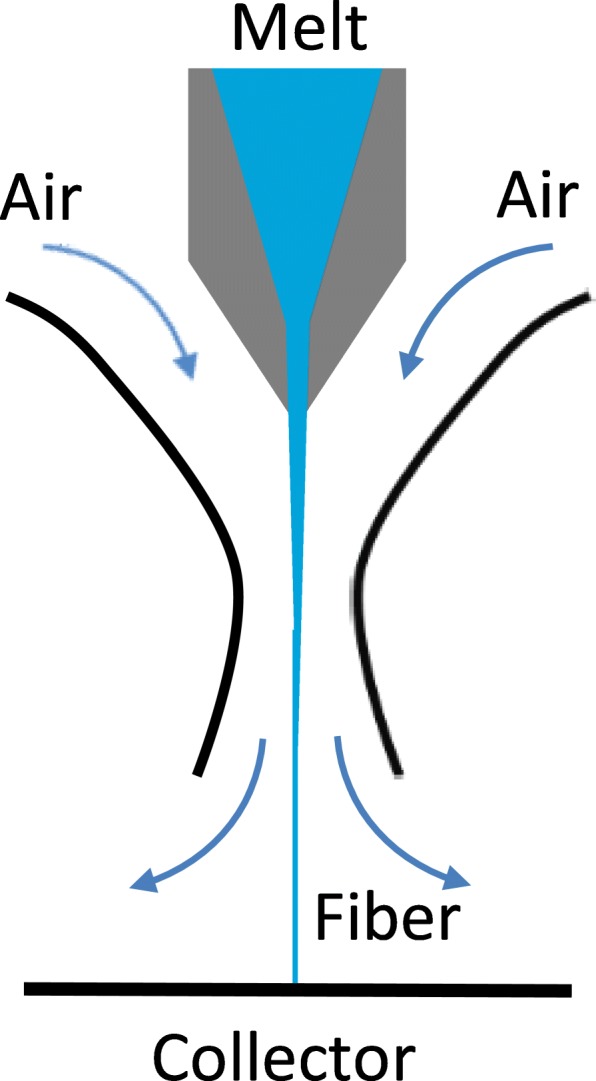
Airflow through the Laval longitudinal nozzle

A derivative of the melt-blowing technique is the Laval spinning method, which also uses an airstream to draw the fiber from the nozzle [64]. However, the shape of the longitudinal Laval nozzle accelerates the air, making the process more efficient than conventional melt-blowing. Furthermore, a cold airstream is used rather than the hot airstream of the conventional method (Fig. 7). The laminar airstream enters the nozzle from the back. The nozzle narrows just beyond the entrance channel for the polymer, which accelerates the air stream and the fiber to supersonic velocity. The main advantage compared to conventional melt-blowing is that the nozzle diameter can be much larger, allowing spinning with a high mass throughput per nozzle [64]. The proprietary Nanoval process is similar to the Laval method but it produces a multitude of smaller-diameter fibers that erupt from the original drawn string when the steadily increasing laminar airflow reaches a particular threshold [65].

**Fig. 8:**
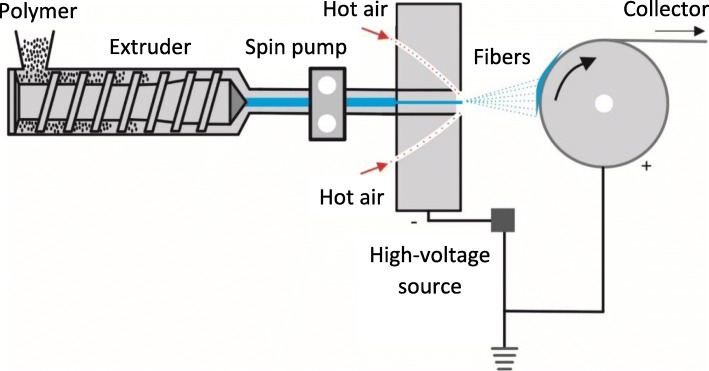
Basic setup of the hybrid melt-electroblowing production method

Electroblowing is essentially a hybrid of electrospinning and blowing [64, 66]. The voltage at the spinning nozzle is sufficient to allow Taylor cone formation. The fiber is then caught by a low-velocity airstream that carries it away from the spinneret in a manner similar to conventional melt blowing. However, in contrast to conventional melt blowing, electrostatic repulsion is the main force that pulls the fiber from the nozzle and the purpose of the airflow is to reduce interference from the electric field of adjacent nozzles, making the process easier to scale up [64]. Like electrospinning, electroblowing has two variants: solution electroblowing and melt electroblowing, the latter illustrated in Fig. 8. In both cases, the airstream also cools down the liquid fiber to solidify it (melt electroblowing) or to dry it and remove the solvent (solution electroblowing).

Flash spinning is a special form of solution spinning, in which the solvent is a hydrocarbon such as butane or isobutene, which would exist as gas under normal atmospheric pressure at room temperature. The spinning solution is maintained under very high pressure at temperatures of 130–500 °C. When the spinning solution is ejected into an environment with a much lower pressure and temperature, the fiber dries immediately [67, 68].

**Fig. 9:**
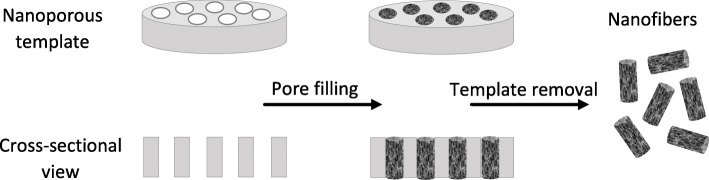
Overview of template synthesis

Other, lesser-used production methods include template synthesis and self-assembly. Template synthesis is suitable for the production of both fibers and tubules [50, 69]. It uses the pores of a host material as a template to control the growth of new materials [57]. For example, polymers can be produced electrochemically by applying a metal layer to a membrane with pores within which the polymers are synthesized [57]. Figure 9 demonstrates the procedure and shows how the fiber diameter and length are controlled by the pore dimensions. [70] Self-assembly is used for the production of nanofibers comprising polypeptides with an intrinsic capacity for self-assembly [71]. The method is based on the spontaneous organization of individual macromolecules into an ordered and stable nanoscale structure [41]. A solution is necessary to create the appropriate environment for the formation of these structures, which have a potential minimum diameter of 3 nm [72]. Although very small diameters can be achieved, this technology is complex and has a low throughput, making it difficult to scale up and thus unsuitable for industrial applications [41].

**Fig. 10:**
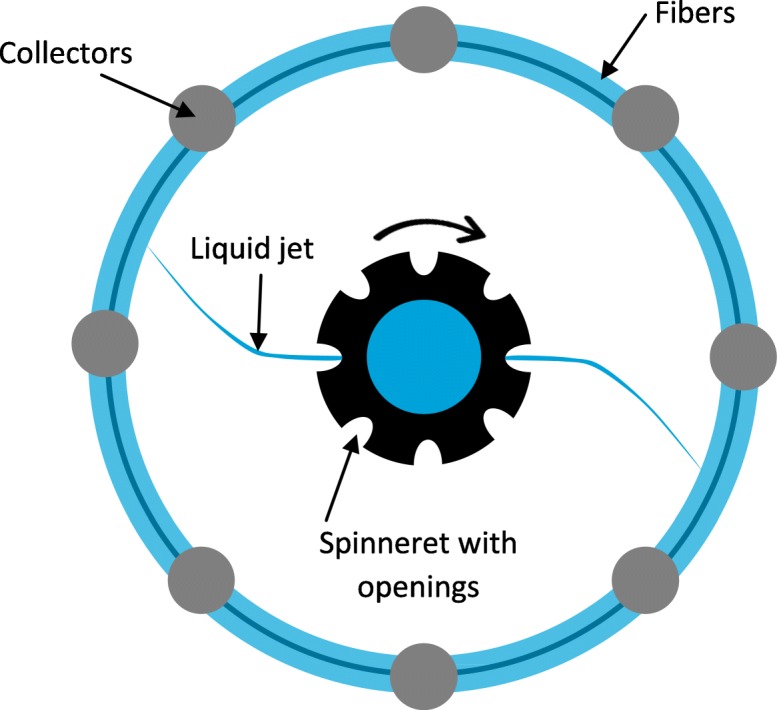
Basic setup of the centrifugal spinning production method

Whereas melt blowing and its variations are easy to scale up, these methods cannot produce oriented fibers. In contrast, the other methods can produce oriented fibers but are hampered by their low throughput. Centrifugal spinning can overcome this challenge by mounting the spinneret on a centrifuge with the nozzles facing outward [73, 74]. When the centrifugal force (dependent on the rotor diameter and angular velocity) is sufficient to overcome the drag caused by the viscosity of the polymer solution or melt, a steady polymer jet streams from the nozzle to the collector [73, 74]. The centrifugal spinning method is shown in Fig. 10. A derivative of this method is split-fiber production, where the nozzle is split into several smaller nozzles to produce narrower fibers or flat bands [75]. The throughput is up to 500-fold higher than conventional solution electrospinning [76]. However, the use of solvents and the strong dependence on the elasticity of the polymer solution and the evaporation rate of the solvents make this process difficult to control [77].

## Development of a new melt-electrospinning prototype for sub-microfibers and nanofibers

### Work leading up to the prototype – state of the art

Electrospinning methods allow the production of single or multiple nanofibers (depending on the number of jets) using a simple apparatus with relatively low setup and operating costs, so electrospinning can be an economically competitive production method [78]. The presence of solvent in the solution electrospinning process adds an expensive recovery step to the overall manufacturing process, and the potential carryover of toxic solvents or solvents with undesirable optical activity makes solution electrospinning unsuitable for medical and filtration applications or the production of optical sensors. Although the high temperature, high viscosity and low conductivity of the molten polymer is a challenge that must be addressed during melt electrospinning [79, 80], the absence of solvent ensures stable jet formation, allowing the direct deposition of micrometer to sub-micrometer range fibers and the reproduction of three-dimensional structures [81–85]. Melt electrospinning is not compatible with non-thermoplastic materials, including biological polymers such as collagen, but is ideal for sparingly-soluble polymers such as PP and polyethylene. Other commonly used polymers include PCL, PU, PLA, and PLGA. [86–92].

In order to produce nanoscale fibers, the polymer delivery rate during melt electrospinning must be significantly lower than during solution electrospinning, which explains the absence of melt electrospinning as an industrial manufacturing method for nanofibers [4]. However, only 2–10% of the liquid processed during solution electrospinning is the polymer (the rest is solvent that evaporates) whereas 100% of the processed liquid solidifies into fibers during melt electrospinning, indicating that the industrial use of this method could be achieved by scaling the process up [4]. Accordingly, recent device innovations, such as multiple-needle and needleless configurations, have demonstrated a roadmap to overcome the low throughput of melt electrospinning, typically in the μg/h range [4]. Prototypes with umbellate nozzles containing 60 spinnerets can achieve maximum product deposition rates of ~ 36 g/h [93, 94]. The largest multi-nozzle spinning device described in the literature thus far features 64 nozzles [95].

### Prototype for the scaled-up melt electrospinning of sub-microfibers and nanofibers

**Fig. 11:**
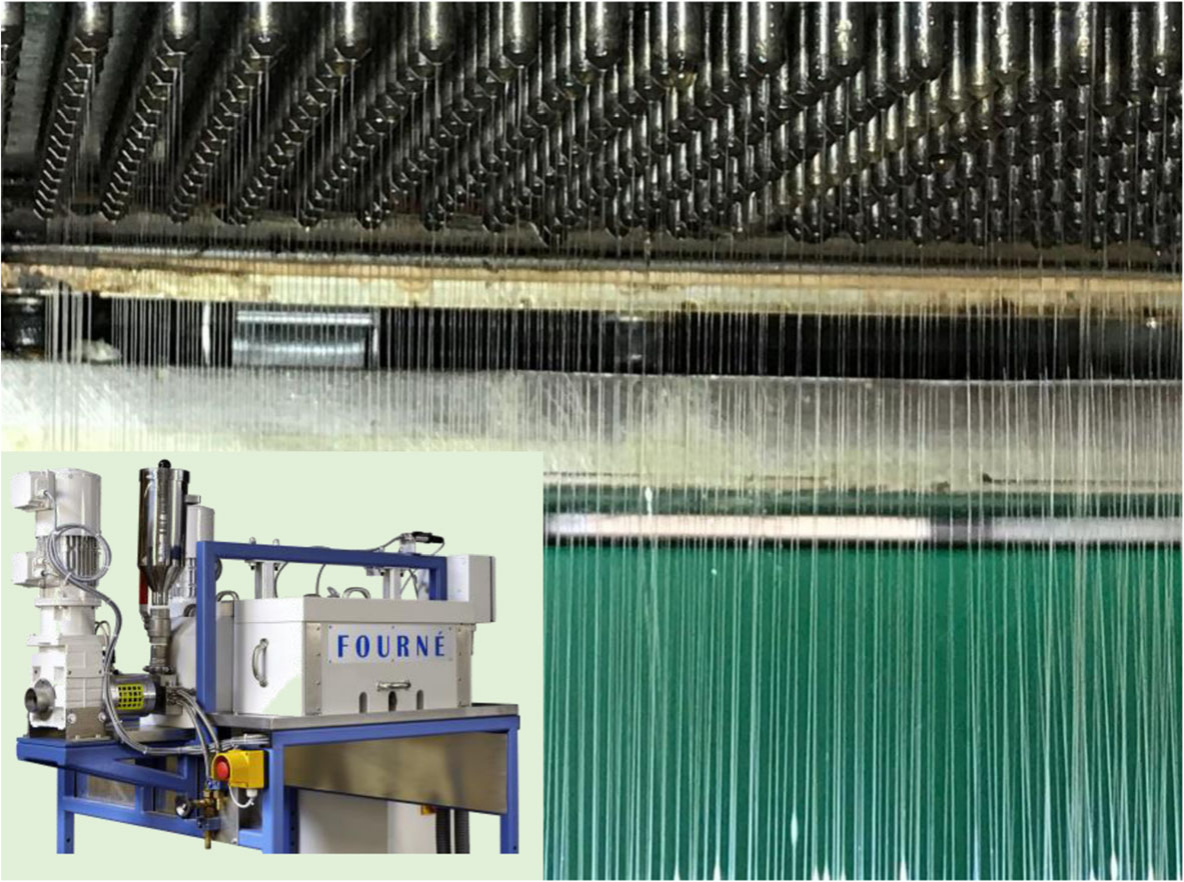
The 600-nozzle melt-electrospinning prototype developed at the AMIBM

The Aachen-Maastricht Institute for Biobased Materials (AMIBM) at Maastricht University has cooperated with Fourné Maschinenbau GmbH (Alfter-Impekoven, Germany) and Pötter-Klima Gesellschaft für Nanoheiztechnik mbH (Georgsmarienhütte, Germany) to develop a functional prototype of a melt-electrospinning device featuring a spinneret with 600 nozzles, which vastly exceeds the capabilities of any state-of-the-art technologies. The nozzle plate of this device is shown in Fig. 11. Each nozzle has a diameter of 0.3 mm and the nozzles are spaced 8 mm apart.

One of the major challenges when scaling up a melt-electrospinning device from a smaller number to a larger number of spinnerets is the uniform distribution of the melt to all nozzles. The low-volume flow of the polymer melt during melt electrospinning may lead to incomplete nozzle filling, resulting in sporadic and unpredictable pressure losses within each nozzle. Inside the prototype nozzle, melt flow has been improved by taking this design consideration into account and introducing a three-plate construction and two symmetrically designed polymer inlets. A distributor plate combined with a finely porous sintered plate ensures the optimum melt distribution and a uniform pressure build-up over the entire nozzle cross-section. A relatively high specific contact load at the sealing line as well as the use of aluminum flanges guarantees the sealing of the nozzle plates. The constant supply of polymer melt is ensured by a speed-adjustable single-screw extruder and spinning pump.

**Fig. 12:**
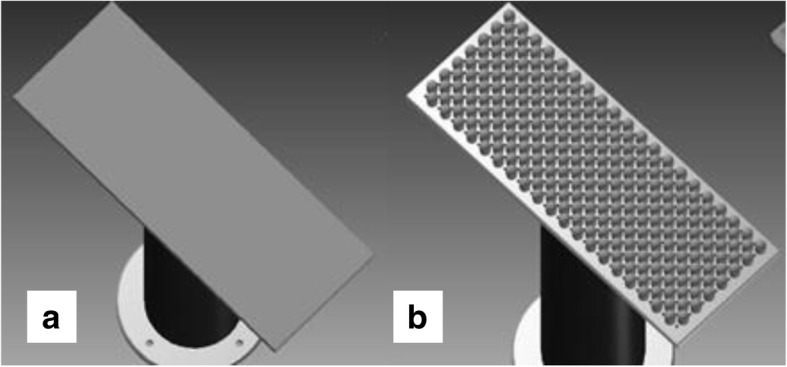
Comparison of (**a**) a conventional collector and (**b**) the novel collector designed for the 600-nozzle AMIBM melt-electrospinning prototype

Another challenge addressed by the new prototype is the tendency for solidified polymer to block the capillaries. The integration of heating elements around the spinneret achieves a uniform polymer melt flow from the nozzles to prevent this common problem during fiber production. A collector with an uneven surface is used instead of a conventional plate collector to facilitate the optimal spreading of the collected fibers (Fig. 12). With the nozzle/collector pairing installed in the prototype, nonwovens can be produced continuously over a width of 340 mm. The collector is connected to an Eltex KNH65 source supplying a positive high voltage (1–60 kV) while simultaneously grounding the spinneret.

**Fig. 13:**
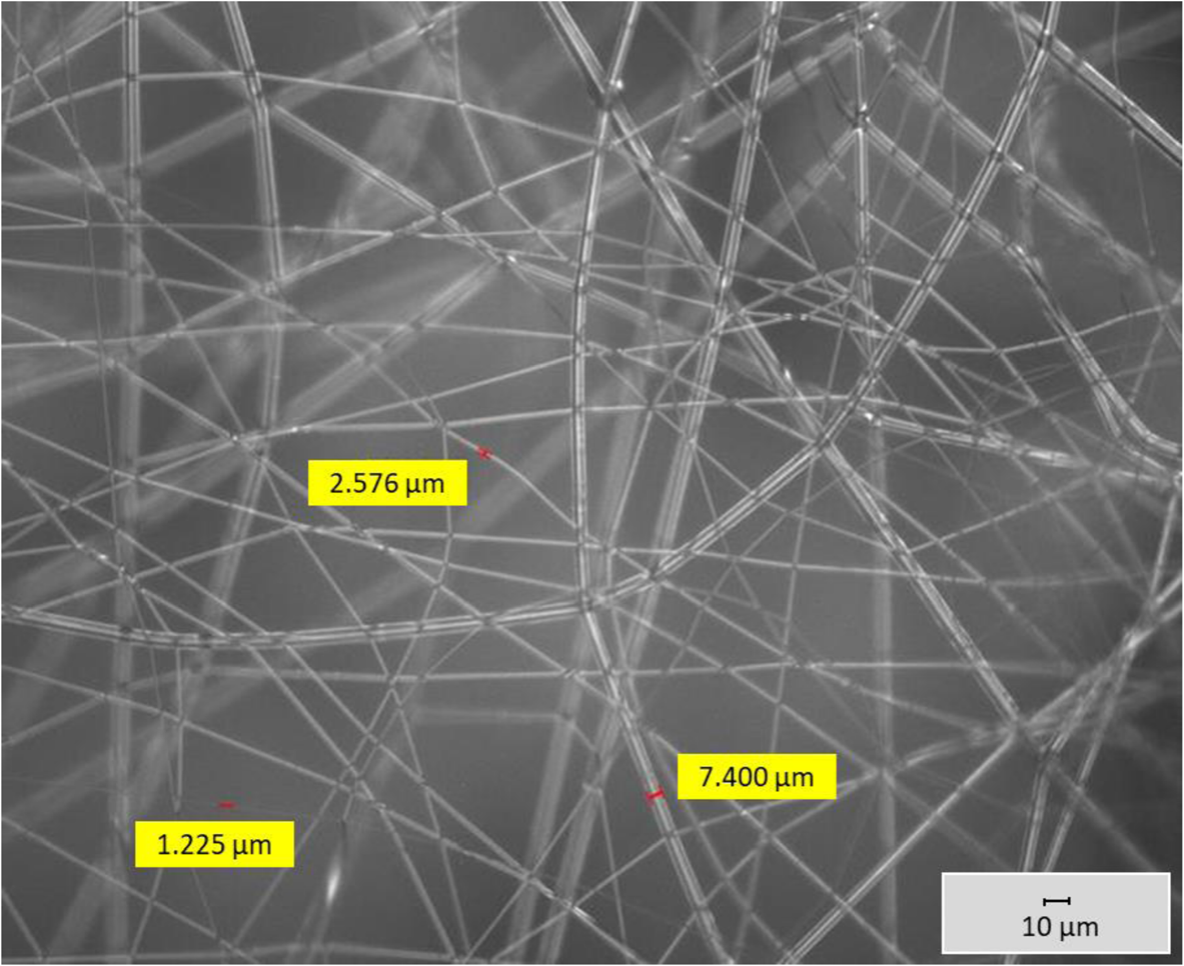
PLA microfibers containing 6% (*w*/w) sodium stearate produced using the 600-nozzle AMIBM melt-electrospinning prototype

Initially, the device was used to produce PP fibers containing conductive additives, and the finest fiber had a diameter of 6.64 μm. This was produced using high-flow PP HL508FB (Borealis AG, Vienna, Austria) containing 2% (*w*/w) sodium stearate (Alfa Aesar, Karlsruhe, Germany). The distance between the collector and the nozzle plate was 11 cm, a positive voltage of 60 kV was applied to the collector, the nozzle was heated to 210 °C and the polymer flow rate was defined by a spinning pump speed of 16 rpm [96]. Having verified the function of the device, we then attempted the production of biobased fibers using Ingeo Biopolymer 6201D, a commercial spinning-grade PLA (NatureWorks LLC, Minnetonka, Minnesota, USA) containing 6% (w/w) sodium stearate. We maintained the distance between the collector and nozzle plate at 11 cm but reduced the nozzle temperature to 190 °C and the spinning pump speed to 2 rpm, yielding fibers ranging from 1.000 to 7.000 μm in diameter (Fig. 13).

## Outlook

Several methods can be used to produce nanofibers and sub-microfibers, but melt electrospinning is among the most promising technologies in terms of fiber structure and the breadth of downstream applications due to the absence of solvents in the manufacturing process. The major drawback of melt electrospinning is its low throughput, resulting in the adoption of solution electrospinning as the principal industrial process technology. Although some attempts have been made to scale up the electrospinning method, an industrial process has yet to be established. At the AMIBM, we have developed a promising, scaled-up melt-electrospinning prototype that bridges the gap between laboratory-scale and pilot-scale manufacturing. Thus far, we have produced PLA fibers ~ 1 μm in diameter, but this was achieved without comprehensive optimization of the apparatus, the process parameters or the polymer substrate and additives. There are many opportunities to improve the performance of the device by adding new features such as a controllable climate chamber around the spinneret to improve jet stretching before the collector, delaying the solidification of the melt and thus producing thinner fibers with uniform diameters. In the future, individually controlled collector tips in a multi-nozzle structure with the writing ability of melt electrospinning could lead to the development of truly innovative microfiber and nanofiber products.

## Abbreviations

AMIBM: Aachen-Maastricht Institute for Biobased Materials
DIN: Deutsches Institut für Normung
MFR: Melt flow rate
PCL: Polycaprolactone
PLA: Polylactic acid
PLGA: Poly(lactic-co-glycolic) acid
PP: Polypropylene
PU: Polyurethane
